# Evaluation of Drug and Herbal Medicinal Promotions on Social Media During the COVID-19 Pandemic in Relation to World Health Organization Ethical Criteria and South African Health Products Regulatory Authority Guidelines in South Africa: Cross-Sectional Content Analysis

**DOI:** 10.2196/58378

**Published:** 2024-09-18

**Authors:** Rujeko Samanthia Chimukuche, Julia Ndlazi, Lucky Thembani Mtolo, Kristien Bird, Janet Seeley

**Affiliations:** 1 Social Sciences Core Africa Health Research Institute Durban South Africa; 2 Division of Infection & Immunity University College London London United Kingdom; 3 Clinical Sciences Core Africa Health Research Institute Durban South Africa; 4 School of Public Health and Nursing University of KwaZulu-Natal Durban South Africa; 5 Department of Global Health and Development London School of Tropical Hygiene and Medicine London United Kingdom

**Keywords:** drug advertising, internet, social media, ethical guidelines, traditional medicine, COVID-19

## Abstract

**Background:**

Consideration of ethics in the promotion of medications is essential to safeguard the health of consumers, particularly during health crises. The World Health Organization (WHO) and the South African Health Products Regulatory Authority (SAHPRA) have established stringent standards to ensure the integrity of pharmaceutical promotions and safeguard public health, including advertisements on the internet and social media platforms. However, the dynamic nature of online advertising poses challenges for monitoring and enforcing ethical standards.

**Objective:**

The study aimed (1) to examine the COVID-19 drug and medicinal promotions across online platforms and social media from 2020 to 2022 in South Africa and (2) to ensure that drug promotions adhere to ethical guidelines outlined by the WHO and SAPHRA.

**Methods:**

A cross-sectional content analysis was conducted to assess drug and medicinal advertisements across various internet and social media platforms. A systematic approach was used to identify and analyze promotional content, focusing on adherence to ethical guidelines outlined by WHO and SAPHRA. Data were collected and analyzed to determine the extent of compliance and identify any potential violations or areas for improvement.

**Results:**

A total of 14 online drug advertisements were included in this analysis. Our findings show that most of the drugs advertised did not meet the regulations and guidelines provided by WHO and SAHPRA. There were omissions about active ingredients, proprietary names, adverse drug responses, precautions, and overdosage and adverse drug reactions. Traditional medicines were not fully consistent with the approved WHO ethical criteria data sheet.

**Conclusions:**

Our analysis highlights the critical importance of ensuring compliance with ethical guidelines in drug promotions on the internet and social media platforms. There is a need for continued vigilance and enforcement efforts to uphold ethical standards and protect the health of the public. Ongoing monitoring and collaboration between national drug regulatory agencies, pharmaceutical companies, and online platforms will be essential for promoting responsible advertising. In addition, safety monitoring and pharmacovigilance systems for herbal medicinal products are yet to be established.

## Introduction

### Background

During the COVID-19 pandemic, there was intense interest in finding potential treatments for the SARS-CoV-2 infection among existing drugs. Several existing medications were publicized as potential treatments for COVID-19 on the internet [1]. Social media and social networks significantly impact communities, and this technology is increasingly becoming an integral part of daily life in modern society [2]. Rapid innovations in information technology are consistently being introduced through various social media and networking websites for communication, information sharing, and entertainment. This increasing dependence on social media and web-based media has been shown to significantly influence behaviors and promote educational learning [2-4].

Faced with a pandemic with no known or approved medications, different untested treatments were advertised and promoted on the internet to the public [5]. In addition, many people were wary of visiting hospitals and relied heavily on social media to obtain information regarding the management of the COVID-19 pandemic [6]. Misinformation spread rapidly from the early days of the COVID-19 outbreak, including falsified information on drug use [7]. Millions of people were exposed to misleading promotions of drugs and services during the pandemic claiming to prevent and cure COVID-19 [7].

Ethics in the promotion of drugs is a critical aspect of the pharmaceutical and health care industries to ensure that the marketing of drugs is conducted in a responsible, transparent, and ethical manner to protect public health, maintain trust, and uphold the integrity of health care systems [8,9]. According to the World Health Organization (WHO), medicinal drug promotion is defined as “all informational and persuasive activities by manufactures and distributors, the effect of which is to induce the prescription, supply, purchase, and/or use of medicinal drugs” [10].

The WHO has laid down ethical criteria for medicinal drug promotion or rational use and has recommended pharmaceutical companies to implement these guidelines, ensuring that advertisements should at least contain a summary of scientific information. Furthermore, WHO indicates that promotional claims for drugs should be truthful, reliable, and not contain misleading or important omissions that may compromise public health [10].

The South African Health Products Regulatory Authority (SAHPRA) ensures that all medicines are registered and advertised in compliance with the Medicines Act. It recognizes that inappropriate promotion and advertisement of medicines contribute to irrational use that potentially brings harm to users [11]. The regulatory body ensures the safety, efficacy, quality, and proper distribution of pharmaceuticals and medical products. SAHPRA evaluates and approves new drugs and medical products for marketing, which involves reviewing extensive data from pharmaceutical companies, including preclinical and clinical trial results, to determine if the product is safe and effective for its intended use [11].

In South Africa, SAHPRA, a statutory body, monitors and regulates the control of medicines in accordance with the Medicines and Related Substances Act 101 of 1965, as amended. Under Regulation 42 for advertising and marketing, an “advertisement” according to the Medicines and Related Substances Act refers to “...any written, pictorial, visual, or other descriptive matter or verbal statement or reference that (a) appears in any newspaper, magazine, pamphlet, or other publication; (b) is distributed to members of the public; or (c) brought to the notice of members of the public in any manner whatsoever, which is intended to promote the sale of that medicine...” [11,12].

However, it should be noted that in South Africa, medications are categorized or scheduled, and as per legal requirements, manufacturing details can be omitted based on the schedule assigned to the medication [11]. Medicine schedules are numbers given to pharmaceutical products based on their benefits and risks. The lower the risk of the product, the lower the scheduled number. Unscheduled medicine on the other hand can be purchased at a pharmacy and this medicine has a schedule of 0. Over-the-counter medicine can be purchased at a pharmacy without a prescription, and this medicine has a schedule of 0, 1, or 2. Prescription-only, controlled substances and strictly controlled substances range from schedule 3 to 8 [13].

Given this background, in this paper, we present an analysis of the ways pharmaceutical drugs were advertised and promoted to treat mild-to-moderate symptoms during the COVID-19 pandemic in South Africa. The research question for this study is: How did the promotion of drugs and medicinal products on online platforms and social media in South Africa between 2020 and 2022 adhere to the ethical guidelines established by the WHO and SAHPRA? The study aims to conduct a thorough analysis of these promotions and assess their compliance with the ethical standards established by WHO and SAHPRA.

The study assessed whether the information provided to the public on drug safety and efficacy was adequate to encourage the rational use of medicinal products. This analysis fills a critical gap in understanding the regulatory compliance and the ethical reliability of drug promotions in a public health crisis. [14]. By evaluating adherence to WHO and SAHPRA ethical guidelines, the study introduces a framework for assessing regulatory compliance in online drug advertising, which is crucial for maintaining ethical standards in social media drug promotions. By integrating both WHO and SAHPRA guidelines, the study provides a dual-perspective analysis that can be used as a model for other regions and regulatory environments. The findings will assist policymakers, regulators, and pharmaceutical companies to improve the ethical standards and effectiveness of drug promotions on web-based platforms.

### Theoretical Framework

In this study, we used the regulation and ethical compliance framework to analyze drug advertisements on social media and their adherence to established ethical and regulatory standards. Regulatory and ethical compliance frameworks have been used in cross-sectional studies to assess health practice adherence to ethical standards [15,16]. Using regulatory frameworks guided by WHO and SAHPRA emphasized accuracy, transparency, ethical considerations, and regulatory compliance. We analyzed data based on these components of the framework. By emphasizing ethical and regulatory compliance in drug promotions, we identified areas that need improvement to protect consumers from harm, promote transparency and accountability in marketing practices, and uphold the integrity of the pharmaceutical industry.

## Methods

### Ethical Considerations

No ethics approval was applied for because according to the Biomedical Research Ethics guidance, certain research projects qualify for exemption from ethics review for example studies on information/data that is already fully in the public domain.

### Study Design

A cross-sectional content analysis was conducted for drug and medicinal promotions on the internet and social media platforms to address whether drugs aligned with the ethical guidelines established by the WHO and SAPHRA. A cross-sectional content analysis provided a snapshot of drug promotional practices on social media, allowing for the assessment of adherence to WHO and SAHPRA ethical guidelines at that specific point in time as done in other studies, such as Vivek et al [17] and Boeson et al [18]. In this analysis, this method was used to assess the ethical and regulatory compliance of drug promotions during the COVID-19 pandemic. Given the dynamic nature of the COVID-19 pandemic and the swift changes in public health communication, a cross-sectional content analysis provided timely insights that could inform immediate regulatory adjustments and public health strategies.

### Study Screening

Drug advertisements were searched for on South African pharmacy websites and social media platforms, such as Facebook (Meta) and Twitter (rebranded as X). Drug and traditional medicine advertisements circulating on WhatsApp (Meta) were also considered. Key search terms included the phrases “COVID-19 treatments in South Africa,” “medications promoted in South African pharmacies during COVID-19,” and “traditional medicines used during the COVID-19 pandemic in South Africa.” Inclusion and exclusion criteria were later used (Textbox 1).

**Inclusion and exclusion criteria.**

**Inclusion criteria**

- Pharmaceutical drugs and substances
- Traditional and herbal-based medicines
- COVID-19 pandemic related
- Promoted in South Africa
- Available from December 2019 to December 2020

**Exclusion criteria**

- Not COVID-19 pandemic related
- Advertisements that were not South Africa based
- Advertisements that had internet and web restrictions placed on them

### Data Charting Process

A total of 14 drug advertisements were extracted to Microsoft Excel and were assessed as per the WHO and SAHPRA guidelines. The WHO ethical criteria [10] used for assessment are listed in Textbox 2.

We used a Microsoft Excel spreadsheet to summarize the data including the source of the advertisements as illustrated in Multimedia Appendix 1.

**World Health Organization ethical criteria for assessment.**

- The names of the active ingredients using either international nonproprietary names or the approved generic names of the drug
- Propriety name of such medicine
- Active ingredient per dosage form or regimen
- Name of other ingredients known to cause problems
- Mention the approved therapeutic uses of the drug
- Side effects and major adverse drug reaction
- Precautions, contraindications, and warnings
- Name and address of the manufacturer or distributor

## Results

A total of 14 drug advertisements were extracted covering the first year of the COVID-19 pandemic. Two authors, RSC and JN, independently assessed the advertisements.

### Promotional Drug Advertisements as Per the Standard Criteria for WHO Ethical Considerations

Most advertisements did not provide full product information. In total, 8 (57%) out of 14 advertisements had no adverse effects mentioned in the wording or illustration. Overall, 10 (71%) out of 14 advertisements did not indicate major adverse reactions that could result from taking the drug. Promotional advertisements omitted the names of the active ingredients, international proprietary names or the approved generic names of the drug, the brand names, and the name and address of the manufacturer or distributors. A total of 13 social media drug promotions did not adhere to ethical guidelines.

#### Names of the Active Ingredients Using Either International Nonproprietary Names or the Approved Generic Names of the Drug

Drug advertisements on hydroxychloroquine, lopinavir, *Lippia javanica* (umsuzwane)*,* and aspirin did not indicate the names of the active ingredients. The other drugs had generic names listed as outlined in Multimedia Appendix 1.

#### Name of Other Ingredients Known to Cause Problems

All the drug advertisements did not provide comprehensive and accurate information about all ingredients. Therefore, there was no indication of drugs that had the potential to cause adverse reactions or interactions.

#### Adverse Effects and Major Drug Reactions

Vitamin C, zinc, rivaroxaban, ivermectin, lopinavir or ritonavir, *Artemisia afra* (umhlonyane), eucalyptus or gum tree extract, and *L javanica* (umsuzwane) did not outline both adverse effects and drug reactions on their promotional advertisements. Favipiravir had adverse effects stated, but no adverse drug reactions.

#### Name and Address of the Manufacturer or Distributor

Five promotional advertisements, namely favipiravir, ivermectin, lopinavir or ritonavir, statins, and aspirin, clearly stated the manufacturer and distributor of the drugs. The rest of the drug advertisements did not specify this detail.

### Traditional Medicine Promoted as Per the Standard Criteria for WHO Ethical Considerations

The wording and illustrations in advertisements for *A afra* (umhlonyane), eucalyptus or gum tree extract, and *L javanica* (umsuzwane) were not fully consistent with the approved WHO ethical criteria data sheet. The text on the traditional medicine advertisements was not fully legible. All 3 substances lacked information on the names of ingredients that may cause problems. The advertisements had no mention of the adverse effects that arise from the use of this traditional medicine. It was observed that major adverse reactions were not mentioned in all 3 advertisements. Warnings were however mentioned for the eucalyptus or gum tree extract, with an indication that it should be used under precaution as directed by a pharmacist

### Promotional Drug Advertisements as per SAHPRA Guidelines for Marketing According to the Medicines and Related Substances Act, 1965

The Medicines and Related Substances Act of 1965 states that drug advertisements should clear proprietary names, the approved name, and the quantity of each active ingredient. According to these guidelines, the drug advertisements that were analyzed lacked sufficient detail. All the drugs, except aspirin, had no proprietary names and active ingredients mentioned in their advertisements. We note that SAHPRA had no guidelines at the time of this study specified for traditional and complementary medicines (Multimedia Appendix 2).

## Discussion

### Principal Findings

Our findings show that most of the drugs advertised did not meet the regulations and ethical guidelines provided by WHO and SAHPRA. The individuals most likely to be aware of WHO, SAHPRA, and South African government regulations are registered pharmaceutical companies, pharmacists, and health care professionals like doctors and nurses who have completed dispensing courses. Influencers and public figures may not be knowledgeable about whether the information they share on their platforms meets legal standards.

The COVID-19 pandemic changed marketing through extensive use of web-based technology. Studies have shown that valuable marketing strategies have been gained during this pandemic, and they can be adopted in the post–COVID-19 pandemic era. A systematic review done on marketing during the COVID-19 pandemic revealed that social media marketing improved the interaction between retailers and consumers [19]. The learnings can be adopted after the COVID-19 pandemic. In this analysis, addressing a key gap in understanding how well drug promotions on social media follow regulations and provide quality information during a health crisis can be adopted for future pandemic crises.

An effective pharmacovigilance system that aligns with WHO, SAHPRA, and national drug policies in South Africa is essential for monitoring and communicating drug safety information to the public. Pharmacovigilance, aimed at minimizing risks and maximizing the benefits of medicinal products, is an important public health tool, as observed in other studies [20,21]. Regulatory bodies should be a crucial part of the national health system, working within the guidelines of clear pharmaceutical policies and legal frameworks [22]. A systematic review of pharmacovigilance systems in resource-limited countries, using the WHO pharmacovigilance indicators, highlighted that strengthening these systems is required with resource and research data consolidation [23]

From this analysis, the most common deviations from the marketing guidelines resulted in the unethical promotion of unapproved or unregistered medicine. There were omissions about active ingredients, proprietary names, adverse drug responses, precautions, and overdosage. Lack of sufficient information about adverse drug reactions may lead to improper use of medications, potentially resulting in harm to patients [24-26]. Furthermore, not knowing about potential drug interactions can lead to dangerous combinations of medications [5].

Complete disclosure is imperative for consumers to make informed decisions about their health care [27]. Being completely transparent ensures that individuals have a clear understanding of the risks involved, helping them make responsible choices for their well-being [28]. Failure to disclose risks and adverse effects, which goes against the WHO ethics guidelines and SAHPRA’s advertising guidelines, involves providing incomplete information about a medicine’s potential adverse effects.

The role of pharmacists in medication management needs to be emphasized and acknowledged. Their vital contribution in assisting individuals in making informed decisions, particularly in the purchase of specific medications, can be improved during health crises. Potential risks arise when there is a lack of health professional guidance when people buy medication. In addition, our findings reveal the lack of regulation of herbal and traditional medicine, multivitamin, and drug supplements in advertising and promotion. With the absence of clear guidelines in South Africa, there is no oversight to verify the accuracy of the information, including ingredients and claims displayed on product containers. Safety monitoring and pharmacovigilance systems for herbal medicinal products are yet to be established [18].

### Policy Recommendations

Based on our findings, we recommend strengthening existing ethical guidelines and regulations established by WHO and SAHPRA to address specific challenges related to web-based and social media drug promotions. This should include updating regulations to cover new digital marketing practices and ensuring that these guidelines are comprehensive and applicable to various online platforms. Furthermore, regulatory frameworks can implement more digitally advanced monitoring systems to regularly review drug promotions on social media and other online platforms for adherence to ethical guidelines. The use of advanced technologies, such as artificial intelligence, to automate the detection of noncompliant content can be useful. Public awareness campaigns to educate consumers about the importance of ethical drug advertising can be created to inform the public on how to identify misleading advertisements and report noncompliant drug promotions. Promoting digital literacy to help consumers critically evaluate online health information and make informed decisions can also be introduced.

Finally, drug and medication regulatory bodies need to be able to enforce stringent guidelines and regulations regarding the inclusion of adverse reaction information in drug advertisements and promotional materials [29]. These regulations should require comprehensive and balanced reporting of these reactions. Disclosing all ingredients, especially those known to cause problems, is essential for patient safety and informed decision-making. Regulatory enforcement, transparency, and education efforts can help ensure that this information is consistently and accurately communicated in drug advertisements and other medication-related materials. Health care providers can also be encouraged to engage in patient-centered care by discussing potential adverse reactions with their patients during medication consultations. This helps patients become active participants in their health care.

### Conclusion

Our cross-sectional content analysis of drug and medicinal advertisements on the internet and social media platforms highlights the critical importance of adhering to ethical guidelines established by the WHO and SAHPRA. This study has shown that continuous monitoring and adherence to these guidelines safeguard public health and ensure the integrity of promotional activities within the pharmaceutical industry, especially during health crises such as the COVID-19 pandemic.

Our analysis, structured around these regulatory frameworks, has uncovered significant gaps in compliance that stress the necessity for more rigorous enforcement of ethical standards. These gaps can lead to the dissemination of misleading or incomplete information, which can have severe consequences for public health.

Concerted efforts from regulatory bodies, pharmaceutical industry, public health organizations, and researchers are needed to ensure that advertising practices are transparent, accurate, and prioritize the well-being of consumers. Further research is needed to explore the underlying factors contributing to noncompliance and to develop effective strategies for improving adherence to ethical regulations. This research should also focus on the impact of digital and web-based marketing practices on public health and evaluate the efficacy of current regulatory frameworks in the digital age. Strengthening these efforts will be vital in maintaining public trust and promoting the rational use of medicinal products.

## Appendix

Multimedia Appendix 1 Analysis of drug advertisements according to the World Health Organization ethical criteria, 2019-2020.

Multimedia Appendix 2 Summary of promotional drugs according to South African Health Products Regulatory Authority Guidelines, 2019-2020.

## Abbreviations

SAHPRA: South African Health Products Regulatory Authority
WHO: World Health Organization
