# Supplementary material for: Evaluation of Drug and Herbal Medicinal Promotions on Social Media During the COVID-19 Pandemic in Relation to World Health Organization Ethical Criteria and South African Health Products Regulatory Authority Guidelines in South Africa: Cross-Sectional Content Analysis
Source: Online J Public Health Inform. 2024 Sep 18;16:e58378. doi: 10.2196/58378 (PMC11447434; doi:10.2196/58378)
Supplement: Multimedia Appendix 1 [file ojphi_v16i1e58378_app1.docx]

**Multimedia Appendix 1.**

| **Name of Drug** | **Name(s) of the active ingredient(s)** | **Proprietary name** | **Active ingredient(s) per dosage form** | **Names of ingredient known to cause problems** | **Mention the approved therapeutic uses of the drug** | **Side-effects** | **Major adverse drug reactions** | **Precautions, contraindications, and warnings** | **Name and address of the manufacturer or distributor** | **Dosage** |
| --- | --- | --- | --- | --- | --- | --- | --- | --- | --- | --- |
| Azithromycin/ Erythromycin | Azithromycin | Zinthromax | N/A | N/A | It is used to treat infections of upper and low respiratory organs (tonsillitis, otitis, sinusitis, pneumonia), urogenital infections intestinal infections, ulcer of stomach and duodenum. | Diarrhoea or loose stools, nausea, abdominal pain, and vomiting, uneven heartbeats, loss of appetite, dark urine, clay-coloured stools, jaundice, fever, rash, red skin, headache. | N/A | There is no evidence if Zithromax affects an unborn baby or excretes in a breast milk. medication should be used in pregnant and breastfeeding women only if expected benefit prevails over potential risk for the baby.  Hypersensitivity to Zithromax and related drugs such as azithromycin or erythromycin. Zithromax is not recommended to take with aluminium- or magnesium- based antacids, such as Mylanta or Maalox as they decrease its absorption in the intestine. | N/A | Take Zithromax tablet with a big glass of water. To prepare a liquid suspension form one dose packet mix one packet with 2 ounces of water, shake and drink at once. Do not use the suspension which was prepared longer than 12 hours ago. |
| Hydroxychloroquine | N/A | N/A | N/A | N/A | N/A | Side effects related to the usage of Hydroxychloroquine vary greatly as they are often seen in different forms with people who have unique underlying issues. For those with cardiac issues, side effects of this drug can include cardiac failure in rare cases that may lead to death. In a similar sense, certain patients have experienced negative neurological side effects like seizures and dizziness as the result of taking this drug. Along with the severe side effects, there are also some minor side effects that can also be experienced. | This drug rapidly absorbs into the body and will start to have issues within the body after just around 30 minutes in certain patients. The most important thing to do when you believe you have overdosed on this medication is to notify your doctor and medical services immediately to get you help. There are a number of issues that arise if you happen to overdose on this drug. Patients will likely need immediate medical attention to ensure they do not experience severe and often deadly cardiovascular and respiratory issues that can arise if too much of this medication is ingested. | Despite the wide range of benefits that are offered by this drug, there are some precautions to be taken before going ahead and using the drug. In particular, those who suffer from gastrointestinal, cardiovascular, or neurological issues. Patients with a history of renal issues and issues that involve the blood should take caution taking this drug as it could trigger issues such as anemia if the body reacts poorly to the drug. These drugs are highly dangerous when exposed to children, so nursing mothers and those with children need to be aware of the risks this medicine poses to younger children. | N/A | Tablets are often administered in sizes of 200 mg serving sizes. Tablets and suspension can be taken with or without food while capsules should be taken on an empty stomach 2 hours before or after a meal. |
| Dexamethasone | Corticosteroids | Decadron | N/A | N/A | N/A | Headache, slowed healing of cuts and bruises ,thin, fragile, or dry skin red or purple blotches or lines under the skin, skin depressions at the injection site increased body fat or movement to different areas of your body inappropriate happiness difficulty falling asleep or staying asleep extreme changes in mood changes in personality depression increased sweating muscle weakness joint pain irregular or absent menstrual periods, hiccups, increased appetite, injection site pain or redness | Sore throat, fever, chills, cough, or other signs of infection, seizures, vision problems, swelling of the eyes, face, lips, tongue, throat, arms, hands, feet, ankles, or lower legs, difficulty breathing or swallowing, shortness of breath sudden weight gain, rash, hives, itching. Cause slow growth in children. Extended use may cause glaucoma or cataracts. | Make the doctor aware of all your allergies. Disclose all your prescription and none prescription medicine. | N/A | N/A |
| Vitamin C | Ascorbic Acid | N/A | Ascorbic Acid 120mg and Organicorange 200mg.Fruit blend (organic apple juice citrus pectin, potassium citrate),apple juice, lemon juice | N/A | N/A | N/A | N/A | When pregnant or nursing and taking other medicine, consult doctor prior to taking the medication. Not to be take by children under 4 years. Keep out of reach of children. Store in cool, dry place and keep tightly closed. Tamper evident with child resistant cap. Do not use if printed safety seal under cap is torn or missing. | N/A | Take 3 gummies daily or as suggested by your health care professional. Chew thoroughly before swallowing. |
| Name of Drug | **Name(s) of the active ingredient(s)** | **Proprietary name** | **Active ingredient(s) per dosage form** | **Names of ingredients known to cause problems** | **Mention the approved therapeutic uses of the drug** | **Side-effects** | **Major adverse drug reactions** | **Precautions, contraindications, and warnings** | **Name and address of the manufacturer or distributor** | **Dosage** |
| Zinc | Essential trace element | [cnt Labs](https://www.takealot.com/all?filter=Brand:cnt+Labs) | N/A | N/A | Colds and flu defence Antioxidant & immune booster. Promotes general well-being. Protects the body against infections & diseases. Helps support the immune system. | N/A | N/A | N/A | N/A |  |
| Favipiravir | Molnupiravir | Lagevrio | Each capsule contains 200 mg molnupiravir | N/A | Lagevrio is an antiviral drug that reduces the ability of SARS-CoV-2, the virus that causes COVID-19, to replicate. It works by changing the genetic material of the virus called RNA, which impairs the ability of the virus to multiply in the body. It is also used to help people with Covid 19 to stay out of hospital. | Nausea, dizziness, diarrhoea, headache | N/A | Do not use whilst pregnant as it may harm the baby. If you can become pregnant, you should use effective birth control during treatment with Lagevrio and for 4 days after the last dose of Lagevrio. Breast-feeding is not recommended during treatment and for 4 days after the last dose of Lagevrio. | The social medwork. | Four 200 mg capsules (800 mg), taken by mouth (orally) every 12 hours for 5 days |
| Rivaroxaban | Xarelto | Rivaxored , Xarelto 20 | Rivaroxaban 20mg | N/A | Xarelto is indicated to reduce the risk of stroke and systemic embolism in patients with nonvalvular atrial fibrillation. Rivoroxaban has been used for the treatment and secondary prevention of acute deep vein thrombosis with or without pulmonary embolism. Xarelto is indicated for the prophylaxis of deep vein thrombosis, which may lead to pulmonary embolism in patients undergoing knee or hip surgery. | N/A | N/A | Premature discontinuation of any oral anticoagulant, including Xarelto, increases the risk of thrombotic events. Epidural or spinal hematomas have occurred in patients treated with Xarelto who are receiving neuraxial anesthesia or undergoing spinal puncture. These hematomas may result in long-term or permanent paralysis. | N/A | N/A |
| Ivermectin | Stromectol | Iverheal | Stromectol 12mg | N/A | Ivermectin is an established antiparasitic that, in South Africa, is registered for use in animals – but not humans. At times, however, the South African Health Products Regulatory Authority (SAHPRA) grants permits for topical ivermectin to be used for patients with conditions such as head lice and scabies. It is used to kill a range of internal and external parasites in livestock and pets, with minimal recorded side effects, giving hope that it can do the same with the coronavirus in humans. | N/A | N/A | Stromectol may cause dizziness, light-headedness, or fainting; alcohol, hot weather, exercise, or fever may increase these effects. To prevent them, sit up or stand slowly, especially in the morning. Sit or lie down at the first sign of any of these effects. Tell your doctor if you have any other parasite infection. Tell your doctor if you have been in an area where Loa loa infection is likely (eg, West or Central Africa).   No not use this medicine if you are allergic to any ingredient in Stromectol. | Trust Pharmacyworld famous pharmacy | Take Stromectol by mouth on an empty stomach with water. Some patients who take Stromectol will need repeated follow-up and retreatment to make sure the infection is cleared up completely. |
| Lopinavir /ritonavir | N/A | AllTera | N/A | N/A | N/A | N/A | N/A | N/A | Mylan | N/A |
| Artemisa Afra/Umhlonyane | Artemisinin | African Warm wood | warm wood 20g | N/A | Used to treat coughs, colds, fever, loss of appetite, colic, headache, earache, intestinal worms, malaria, sweaty palm and a blocked nose. | N/A | N/A | N/A | N/A | Add a quarter cup of dried leaves to a cup of boiling water, allow the infusion to draw for 10 minutes then strain the mixture and add some honey to sweeten the tea. |
| Eucalyptus / Gumtree extract | Eucalyptus | Hercules | N/A | N/A | N/A | N/A | N/A | Keep out of reach of children. Use only as directed by a medical practitioner or pharmacist. | N/A | N/A |
| Umsuzwane/Lippia Javanica | N/A | N/A | N/A | N/A | It is used to rub bodies with the leaves to protect them against lightning. Umsuzwane is also used for coughs, colds, influenza and headaches | N/A | N/A | N/A | N/A | N/A |
| Statins | Simvastatin | Zocor | Simvastatin, 5mg-10mg-20mg-40mg | N/A | Used to lower high cholesterol and triglycerides in certain patients. Reduces the risk of heart attack, stroke and death due to corinary heart disease. Reduces need for medical procedures to open blocked blood vessels. Reduce chest pain caused by angina. | Constipation, heart burn, stomach gas, pain | Allergic reaction such as skin rash, itching or hives, swelling of the face, lips or tongue. Dark urine, fever, joint pain, muscle cramps or pain. Redness, blistering, peeling or loosening of the skin, including inside the mouth, trouble passing urine or change in the amount of urine, unusually weak or tired yellowing of the eyes or skin | Do not take this product when you are taking the following treatment-  • medicines for fungal infections like itraconazole, ketoconazole •nefazodone • other medicines for high cholesterol •red yeast rice • some antibiotics like clarithromycin, erythromycin, telithromycin • some medicines for HIV infection like amprenavir, indinavir, ritonavir Consult paediatrician for use in children. Do not share this medicine with others. | Online Pharmacy in South Africa. | Taken by mouth with a glass of water-taken with or without food-regular intervals. If you have overdosed contact poison control centre or emergency room. |
| Asprin | N/A | Bayer | Asprin 300mg | N/A | Bayer Aspirin 300mg 30 Tablets brings you effective relief of pain – from mild to moderate – and can be used for headaches, toothache, menstrual cramps, neuralgia, muscular pain and throughout winter to help with pain associated with colds, flu and fever. | Stomach pain or discomfort, indigestion or heartburn. Nausea or vomiting. | Unusual bleeding or bruising. Black stools or severe diarrhoea. Ringing in the ears, severe headache, dizziness or drowsiness. Confusion, changes in vision or changes in behaviour. Excessive sweating or increased thirst. Skin rash or itching. Any difficulty in breathing. Swelling of the eyelids, face or lips. | Keep out of reach of children. Do not use continuously for more than 10 days without consulting your doctor. Before using this medicine: Inform your doctor and pharmacist about alcohol or any other medicines you are taking, especially other blood thinning agents, other pain killers or medicines used for arthritis, sugar diabetes or gout. Do not use if allergic to aspirin or pregnant, intend on becoming pregnant or breast feeding. Inform your doctor if you have ever had an ulcer, have a tendency to bleed, have kidney problems, diabetes, gout, asthma or other allergies. Do not use aspirin in children or teenagers unless directed to do so by your doctor. Do not take aspirin a week before you have any surgery done, including dental surgery Store Bayer Aspirin® at or below 25 degrees C and out of reach of children. Return any unused or expired medicine to your doctor, pharmacist or health care professional for safe disposal. Do not take Bayer Aspirin® tablets that smell strongly of vinegar. | Clicks pharmacy | Adults: one to three tablets should be taken when necessary. The dose may be repeated every four hours but do not take more than 12 tablets during any 24 hour period. Children over the age of 12: one to two tablets. The dose may be repeated every four hours but do not take more than 10 tablets during any 24 hour period. The tablets should be taken with a full glass of water after meals or with food. Do not use continuously for more than 10 days without consulting your doctor. |
