# Supplementary material for: Evaluation of Drug and Herbal Medicinal Promotions on Social Media During the COVID-19 Pandemic in Relation to World Health Organization Ethical Criteria and South African Health Products Regulatory Authority Guidelines in South Africa: Cross-Sectional Content Analysis
Source: Online J Public Health Inform. 2024 Sep 18;16:e58378. doi: 10.2196/58378 (PMC11447434; doi:10.2196/58378)
Supplement: Multimedia Appendix 2 [file ojphi_v16i1e58378_app2.docx]

**Multimedia Appendix 2.**

| **Medicine Trade Name** | **Medicine Active ingredient** | **Scheduling Status** | **Registered with SAHPRA (Binary)** | **Source of advertisement** | **SAHPRA Advertisement guidelines violated? (binary)** | **Legal framework variation comment (s)** |
| --- | --- | --- | --- | --- | --- | --- |
| Azithromycin/ Erythromycin | Azithromycin | 4 | Yes | https://africarx.co.za/zithromax/ | No active ingredients per dosage form and proprietary names of drugs were mentioned in the advert. | Prescription required when purchasing |
| Hydroxychloroquine | Hydroxychloroquine | 1 | Yes | https://directmeds.co.za/product/hydroxychloroquine/ | Active ingredient mentioned. No manufacturer | Prescription required when purchasing |
| Dexamethasone | Dexamethasone | 6 | Yes | Dexamethasone \| South Africa Drug Approvals \| Medicines Control Council \| PharmaCompass.com | The active ingredient which is dexamethasone is not specified on the advert. Therapeutic uses and dosage not clarified. No manufactures or distributor. | Should not be able to purchase it without a prescription from doctor |
| Vitamin C |  |  | No | https://clicks.co.za/clicks_vitamin-c-500mg-100-tablets/p/53535 | No ingredients indicated. | Vitamin C is a vitamin (and the active ingredient) and comes in many combinations and product names. No regulatory body in SA checks that what is advertised is true. |
| Zinc |  | 3 | Yes | https://www.takealot.com/zinc-25mg-30-tablets/PLID70335759?fbclid=IwAR3UNhLmAh8DW2_xXJpcW3amD4gLSdjqhAlXl__mRRzcaLbLW2fCIsMoLo0  https://zinplex.co.za/products/new-zinplex-zinc-dispersible-tablets | The adverts did not clarify most guidelines including mention of active ingredients, side effects and warnings |  |
| Favipiravir |  |  | No | https://thesocialmedwork.com/lagevrio-molnupiravir  https://twitter.com/SAHPRA1/status/1494288748657455107?ref_src=twsrc%5Etfw%7Ctwcamp%5Etweetembed%7Ctwterm%5E1494288748657455107%7Ctwgr%5E%7Ctwcon%5Es1_&ref_url=https%3A%2F%2Fbusinesstech.co.za%2Fnews%2Ftrending%2F560002%2Fregulator-authorises-covid-19-pill-for-south-africa%2F | 17 February 2022: SAHPRA authorised, with conditions, the importation of molnupiravir 200mg capsules (“LAGEVRIO”), to be provided by MSD (Pty) Ltd, in terms of section 21 of the Medicines and Related Substances Act, 1965. This authorisation is for a limited quantity of “LAGEVRIO” and is initially limited to a period of six (6) months. | This is not registered in South Africa and can only be imported on a section 21. This is done under the supervision of the prescribing doctor and reporting to SAHPRA must be done during the time that the patient uses the medication. It would be scheduled if available in South Africa. |
| Xarelto Bayer | Rivaroxaban | 4 | Yes | https://www.pharmacompass.com/south-african-mcc-mpr-drug-database/rivaroxaban | Promotional adverts on the drug rivaroxaban lacked majority of guidelines that make it ethically sound according to WHO. No manufacturer, no side effects, contraindications, and warnings mentioned. | Should not be able to purchase it without a prescription from doctor |
| Ivermectin |  |  | Not for human consumption | https://www.facebook.com/ClicksSouthAfrica/posts/the-fda-approved-drug-ivermectin-inhibits-the-replication-of-sars-cov-2-in-vitro/4003298619723006/  https://www.indiamart.com/proddetail/iverheal-ivermectin-tablets-23819500955.html https://twitter.com/SAHPRA1/status/1531240270313971712?ref_src=twsrc%5Etfw%7Ctwcamp%5Etweetembed%7Ctwterm%5E1531240270313971712%7Ctwgr%5E%7Ctwcon%5Es1_&ref_url=https%3A%2F%2Fewn.co.za%2F2022%2F05%2F30%2Fivermectin-no-longer-allowed-to-treat-covid-19-in-south-africa https://dir.indiamart.com/search.mp?ss=ivermectin+tablets&prdsrc=1&src=as-rcnt%3Apos%3D4%3Acat%3D-2%3Amcat%3D-2  http://pharmstore.com/categories/Antibiotics/Stromectol/?id=1978 | The studies that suggested potential efficacy of Ivermectin in the prevention and treatment of COVID-19 and which motivated the adoption of the Programme have since been retracted. The WHO advised that this drug be used in the context of clinical trials. Issued in Pretoria 30 May 2022.  Ivermectin use on human was discontinued in SA after two large clinical trials conducted in 2021 stating that potential efficacy of Ivermectin has been limited |  |
| Kaletra | Lopinavir/Ritonavir | 4 | Yes | https://www.cmmediclinic.com/buy-lopinavir-ritonavir-online.html |  |  |
| Artemisa/Afra/ Umhlonyane |  |  | No | Medico Herbs Artemisia Afra African (Wormwood) 400mg 60 Tablets \| hiVibe.co.za  https://seedsandplants.co.za/products/dried-herbs-and-spices/dried-african-wormwood-20g/ | The promotional advert lacks detailed information on the adverse reactions that may arise, side effects and names of manufacturers. No SAHPRA guidelines are provided on the use of African traditional medicine |  |
| Eucalyptus/ Gumtree Extract |  |  | No | https://clicks.co.za/clicks_eucalyptus-oil-50ml/p/81189?gclid=EAIaIQobChMIzebZzbat-AIVqujtCh0bgg5-EAQYAiABEgINCvD_Bw | The advert does not have most guidelines specified for complementary medicines |  |
| Zocor | simvastatin | 4 | Yes | http://www.medicair.co.za/tab/order-zocor-south-africa.html. | Advertisement did not clarify any ingredients that could cause problems. | This medicine is available only with your doctor's prescription |
| Umsuzwane/Lippia/Javanica | N/A | N/A | No | **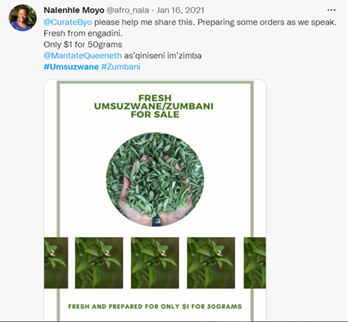** | No SAHPRA guidelines are provided on the use of African traditional medicine. The advertisement did not have much detail | N/A |
| Bayer | Aspirin | 0 |  | https://clicks.co.za/bayer_aspirin-300mg-30-tablets/p/3551 | Promotional advert had most of the SAHPRA guidelines |  |
